# Systematic Reviews and Meta-analysis: Principles and Practice

**DOI:** 10.31729/jnma.3986

**Published:** 2019-02-28

**Authors:** Badri Man Shrestha

**Affiliations:** 1 Sheffield Kidney Institute, Sheffield Teaching Hospitals NHS Trust, Sheffield, United Kingdom

For providing safe and high-quality care to the patients, health care policies are being formulated as practice guidelines, which are based on clear and comprehensive summaries of information collated through systematic reviews and meta-analysis of relevant literature. Clinical trials undertaken to investigate the effects of interventions in the management of clinical conditions report wide variations in the outcomes, which can pose significant difficulties to the clinicians in selecting best modality of intervention for their patients. In the current age, evidence-based practice with the adoption of practice guidelines has become mandatory in every field of medicine and it is important to appreciate the pivotal role played by systematic reviews in our professional lives. This editorial summarises the principles of systematic reviews and meta-analysis, interpretation of their results and application in clinical practice.

Systematic reviews and meta-analysis are the highest quality evidence (level 1) on a research topic because their study design reduce bias and produce more reliable findings. It is a misconception that systematic reviews and meta-analysis are the same and the terms are used interchangeably. A systematic review is a detailed, systematic and transparent way of gathering, synthesising and appraising evidence to answer a well-defined clinical question. On the other hand, a meta-analysis is a statistical procedure for combining numerical data from multiple separate studies and is always conducted in the context of a systematic review. Two papers published by the author cite the examples of systematic reviews and meta-analysis.^1,2^

As systematic reviews are carried out to generate answers to focussed questions about health care and related issues, a structured approach should be adopted in framing questions by using components such as populations, interventions, comparator, outcomes, timing and setting (PICOTS). The proposed review should be registered with a clinical trial register database at a very early stage, which can provide supports in conducting the review. The proposed methodology should be published in advance in the form of a protocol. Thorough literature search and screening of the search relevant to inclusion criteria should be done by using the Preferred Reporting Items for Systematic Review and Meta-Analyses (PRISMA) guidelines. Any discrepancies in eligibility judgements should be resolved by discussion between the authors. The methodological quality of the included studies “Newcastle-Ottawa scale” (NOS)^3^, grades of evidence (Oxford Centre of Evidence-Based Medicine -Level of Evidence)^4^ and potential for bias (Cochrane Collaboration's tool for assessing risk of bias by Higgins)^5^ should be assessed by using appropriate methods. Data synthesis by analysis of data employing appropriate statistical methods is carried out. The most commonly used statistical software for systematic review and meta-analysis is RevMan 5.3.5 software (Nordic Cochrane Centre, The Cochrane Collaboration, 2014), which is recommended by Cochrane Collaboration Database System.^6^

Meta-analysis should include assessment of heterogeneity between studies by using Q (heterogeneity c^2^) and the I^2^ statistics. In presence of significant heterogeneity (I^2^>50%), random effect model, and for homogeneous studies, a fixed effects model and the Mantel-Haenszel method are adopted. The results are reported for smaller event rates (0-1) by using the Peto method; an odds ratio (OR) for dichotomous outcomes, and weighted mean difference (WMD) for continuous outcomes. Summary estimates and 95% confidence intervals are calculated, and overall effects are determined by the using Z-test. P<0.005 was considered significant. Forest plots are drawn based on these results. The minimum number of studies considered appropriate for display of forest plot is two. A forest plot displays results (that is, estimates of intervention effect) and confidence intervals for individual studies and meta-analyses.^7^ Finally, the quality of each conclusion is assessed by the Grading of Recommendations, Assessment, Development and Evaluation (GRADE) tool (GRADE pro GDT, Cochrane Community, UK).^8^ The results of the review should be published for dissemination and the review should be updated in the future if new evidences are produced.

There are several sources of systematic reviews and guidelines available online. The most widely used Cochrane Library has databases of published and ongoing systematic reviews which include; the Cochrane Database of Systematic Reviews (CDSR), Database of Abstracts of Effects (DARE), Health Technology Assessment (HTA) Database, and Collaborative Review groups (CRGs).^9^ They are extremely valuable sources of high level evidence in medicine. Guidelines make recommendations to each type intervention, where strength is indicated as Level 1 (we recommend), Level 2 (we suggest), or not graded, and the quality of the supporting evidence is shown as A (high quality), B (moderate quality), C (low quality), or D (very low quality).^10^

It is important to appreciate that the quality of the conclusions derived from any systematic review and meta-analysis are influenced by the level of evidence provided by individual studies included in the review. A meta-analysis including adequately powered prospective randomised controlled trials provides high quality evidence, whereas inclusion of retrospective cohort and inadequately powered prospective studies provide low quality evidence in support of the questions under investigation. Therefore, it is important to assess the strength and weakness of individual studies included in the analysis, interpret the conclusions with caution before accepting the conclusions for writing practice guidelines and adopting them in the clinical practice.^11^ It is important for practicing clinicians to have sound understanding of the principles of systematic reviews and meta-analysis and their clinical implications, which can be achieved by attending relevant courses and workshops and being part of a team with interest in the subject.

## Conflict of Interest

**None.**
